# The Unexpected Identity of *Tympanis vagabunda*

**DOI:** 10.3390/life13030661

**Published:** 2023-02-28

**Authors:** Luis Quijada, Hans-Otto Baral, Donald H. Pfister

**Affiliations:** 1 Department of Organismic and Evolutionary Biology, Farlow Library and Herbarium, 22 Divinity Avenue, Cambridge, MA 02138, USA; 2 Independent Researcher, Blaihofstr. 42, 72074 Tübingen, Germany

**Keywords:** fungi, pathogens, phylogeny, Rutstroemiaceae, saprobes, Tympanidaceae

## Abstract

*Tympanis* species (Leotiales) are plant pathogens distributed mostly in northern temperate ecosystems. The diversity and identity of some species remains unclear. *Tympanis vagabunda*, found in Sicilia (Italy) on dry twigs of *Rosa*, *Rubus*, and *Pistacia*, is one example of an obscure and poorly known species. During the study of its type specimen in S, which contained one twig with a wood anatomy fitting neither of the three mentioned hosts, the microanatomic structures indicated that it belongs to the genus *Rutstroemia* (Helotiales). To investigate its identity, the types of *R. fruticeti*, *R. juniperi*, *R. urceolus*, and *R. longiasca* were studied for comparison. The species for which molecular data were available were included in a dataset that contained identified species of *Rutstroemia*, along with other select species from the families Rutstroemiaceae and Sclerotiniaceae. *R. fruticeti*, a saprobe frequently reported from *Rubus fruticosus* in Europe, is found to be a later synonym of *T. vagabunda*, and the combination *Rutstroemia vagabunda* is proposed. *R. juniperi* is an infrequently reported European species on twigs of *Juniperus* and is morphologically hard to distinguish from *R. vagabunda*; available molecular data support its recognition as a distinct species. *R. longiasca* differs from *R. vagabunda* in its black apothecia, smaller asci, and narrower ascospores. *R. urceolus* differs from *R. vagabunda* in having black apothecia and smaller inamyloid asci, and excipulum at the flanks and margin is composed of dark-walled hyphae.

## 1. Introduction

Species of *Tympanis* Tode (Leotiomycetes, Leotiales, Tympanidaceae) are worldwide plant pathogens that are mostly distributed in northern temperate ecosystems. The diversity of the genus is unclear due to the application of different species concepts. Several of the ca. 140 species listed in official databases under *Tympanis* have been transferred elsewhere. Stable nomenclature within a genus depends on clear species delimitations. Stability is generally not an issue in genera with few species that are easily recognizable, but it can be a problem in highly diverse genera with many recognized species. *Tympanis* is a good example of such a large genus. This plant pathogen has been reviewed twice in the last century [1,2]. These treatments do not agree in species concept or number of species. Groves [1] described 35 species in the genus, 19 of which he proposed as new. To delimit species, he used plant hosts as a character, as well as a few macromorphological features (cluster-like growth, apothecial dimensions), but mainly focused on the morphology and size of asci, ascospores, and ascoconidia [1]. In contrast, Ouellette and Pirozynski [2] paid special attention to ascospore germination patterns within the asci. In their concept, host plants were unimportant, resulting in several changes to Groves’ treatment. They accepted 27 species, referred 10 taxa to synonymy (*Tympanis abietina* J.W. Groves, *T. acericola* J.W. Groves, *T. columnaris* (Wallr.) Höhn., *T. diospyri* J.W. Groves, *T. hansbroughiana* J.W. Groves, *T. hypopodia* Nyl., *T. juniperina* (Sacc.) Mussat, *T. piceae* J.W. Groves, *T. sorbi* J.W. Groves, and *T. syringae* Fuckel), and erected 6 new species (*T. alpina* Ouell. and Piroz., *T. heteromorpha* Ouell. and Piroz., *T. grovesii* Ouell. and Piroz., *T. neopithya* Ouell. and Piroz., *T. pulchella* Ouell. and Piroz., and *T. pseudoalnea* Ouell. and Piroz.). Today, there is still disagreement over the number of species in the genus—27 or 64 species [3,4].

During the revision of *Tympanis*, the first author made a list of species that required re-examination. The idea was to clarify the identity of these species based on type studies and investigate the question of misplaced species—that is, species that belong in other genera. The list of specimens to be studied includes 54 species recognized by Groves [1] and Ouellette and Pirozynski [2]; some species were not evaluated by these authors, i.e., those as “not seen”, like *Tympanis antarctica* Speg., *T. buchsii* (Henn.) Rehm, *T. bupleuri* Velen., etc. The largely unpublished revision of these types specimens by the first author has resulted in clearer circumscriptions of taxa, including the question of whether they are species properly placed in *Tympanis*. For example, the revision of *T. buchsii* resulted in its placement as a synonym of *T. confusa* Nyl. [5]. In contrast, Groves [1] and Ouellette and Pirozynski [2] categorized some species as doubtful or excluded. One of these species was *Tympanis vagabunda* Pass. and Beltrani [6]. It was found in Sicilia (Italy) on dry twigs of *Rosa*, *Rubus*, and *Pistacia*, and later combined by Saccardo [7] as *Cenangium vagabundum* (Pass. and Beltrani) Sacc. Groves [1], who did not study the type specimen, followed Saccardo’s opinion, whereas Ouellette and Pirozynski [2] did not mention this species at all. Until recently, *T. vagabunda* has been listed in Index Fungorum and Mycobank as the current name of an accepted species, with *Cenangium vagabundum* as a nomenclatural synonym. Our morphological study of the type of *T. vagabunda* revealed its obvious conspecificity with *Rutstroemia fruticeti* Rehm, whereas other similar *Rutstroemia* P. Karst. species growing on the same hosts (*Rosa*, *Rubus*) appear to represent a distinct species. The aim of this work is to clarify the identity and generic relationship of *Tympanis vagabunda* and explore the relationships among *Rutstroemia* species.

## 2. Materials and Methods

### 2.1. Taxonomical and Morphological Comparison

The type material consisted of three apothecia. Given the scarcity of fruitbodies, no molecular studies were attempted to extract DNA from the fruitbodies of the type of *Tympanis vagabunda* housed in the Swedish Museum of Natural History (S); only a morphology study was done to verify its identity and possible affiliation or misplacement in the genus *Tympanis*. This study led us to conclude that *T. vagabunda* was indeed a *Rutstroemia* species; therefore, a bibliographic review of *Rutstroemia* was done to find the most morphologically and ecologically similar species to *T. vagabunda*. All *Rutstroemia* species names found in [8] were included in this search. Literature was found by using Harvard University’s online library catalog (HOLLIS). Data about locality, ecology, and morphology of the most similar species are compared. The type specimens of those species—*R. fruticeti* in The New York Botanical Garden (NY), *R. juniperi* K. Holm and L. Holm in NY, *R. longiasca* (Cavara) W.L. White in Farlow Herbarium (FH), and *R. urceolus* (Sacc.) W.L. White in FH—were studied morphologically. The techniques for apothecia examination are based on Quijada [9]. Freehand apothecial sections were made under a stereomicroscope (Leica EZ4) or embedded in Arabic gum and sectioned a ca. 20 µm on a freezing microtome. The sections were studied with a compound microscope (Motic B1). Microphotographs were taken with a USB Moticam 2500 camera. The biometrics in the descriptions were done with 95% confidence intervals calculated for each morphological feature using SPSS 15.0 (SPSS Inc., Chicago, IL, USA). Measurements are given as follows: (smallest single measurement–) smallest mean—largest mean (–largest single measurement). The smallest and largest means are based on ≥20 measurements for asci, paraphyses, and excipular cells, and 70 measurements for ascospores. Color terminology refers to [10]. Abbreviations: * = living state; † = dead state; CR = aqueous Congo red; H_2_O = tap water; KOH = potassium hydroxide; LBs = lipid bodies; MLZ = Melzer’s reagent.

### 2.2. Molecular Analyses

Sequences of identified species of *Rutstroemia*, as well as other genera and species in the families Rutstroemiaceae and Sclerotiniaceae, were used to explore their phylogenetic relationships. The sequences were retrieved from GenBank after comparing several publications that include these two families [11,12,13,14,15,16]. Two rDNA regions (ITS and LSU) were used to conduct the phylogenetic analyses, and the final dataset contains a combination of taxa not previously published. The 109 sequences (Table 1) were aligned using the L-INS-i algorithm for ITS and the G-INS-i algorithm for LSU with MAFFT [17]. Gblocks was used to identify and remove ambiguously aligned regions [18] using the parameters: minimum number of sequences for a conserved or flanking position = 21; maximum number of contiguous non-conserved positions = 8; minimum length of a block = 5; and gaps in an alignment column allowed in up to half the number of included sequences. The GTR+G substitution model was identified as the optimal model using JModelTest [19] based on the Akaike information criterion [20]. Bayesian inference (BI) analyses were performed using Geneious (v.6.1.7) following Quijada et al. [21]. The artwork for the phylogenetic tree was prepared in Adobe Illustrator CS5.

## 3. Results

### 3.1. Morphology

**Figure 1:**
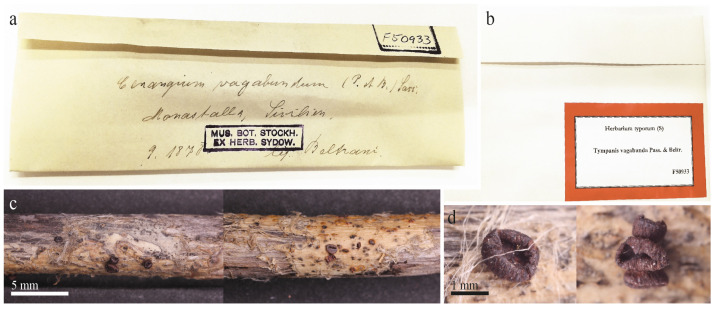
The type specimen of *Tympanis vagabunda*: (**a**) original packet with the handwritten name and locality information; (**b**) cover packet from the herbarium S with collection number F50933; (**c**) the single twig with several apothecia; (**d**) closeup showing apothecia (in dry state).

**Figure 2:**
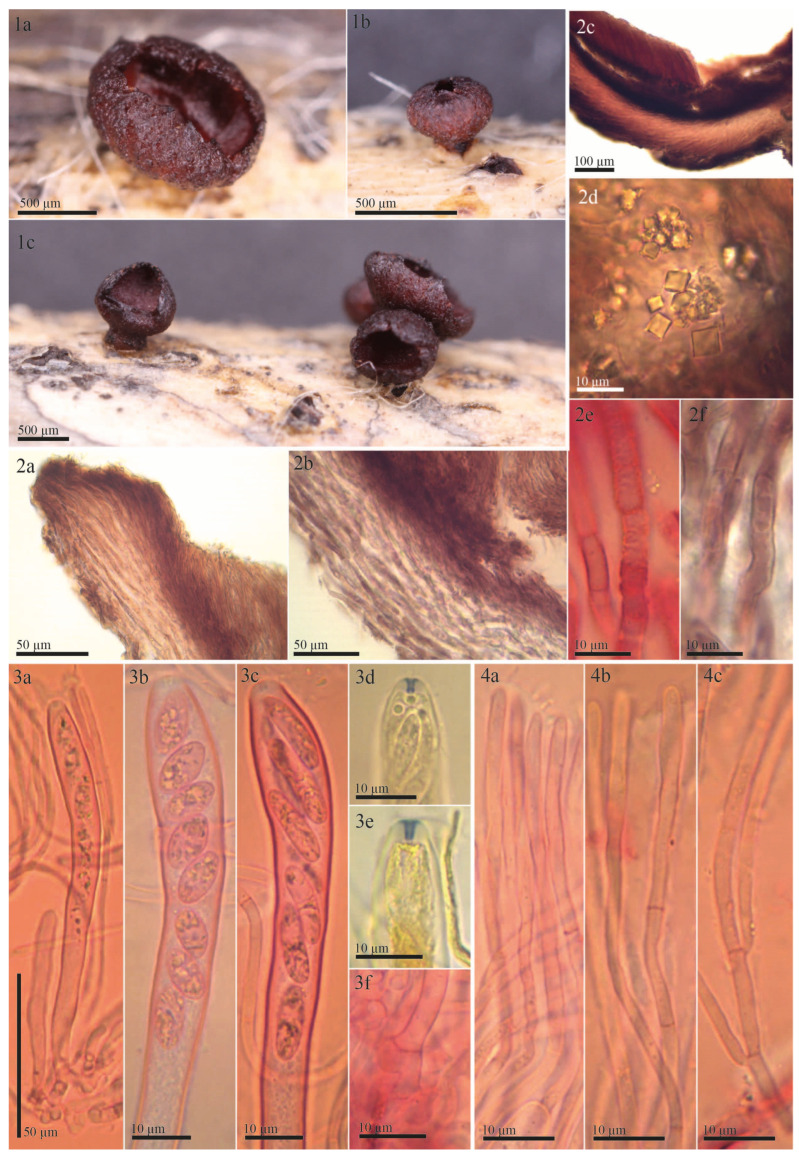
Morphological details of the type of *Rutstroemia vagabunda* (≡ *Tympanis vagabunda*): (**1a**–**1c**) rehydrated apothecia; (**2a**–**2f**) sections showing excipular tissues; (**2a**) ectal excipulum at margin; (**2b**) ectal excipulum at lower flanks; (**2c**) transverse section showing different layers of excipulum and hymenium; (**2d**) octahedral crystals in ectal excipulum; (**2e**,**2f**) cortical hyphae loosely woven and embedded in refractive hyaline gel and walls with irregular patches of dark exudate that produces a banded aspect; (**3a**–**3f**) asci; (**3a**–**3c**) asci with immature and mature ascospores, showing variation in spore morphology; (**3d**,**3e**) amyloid apical ring; (**3f**) ascus base with crozier; (**4a**–**4c**) paraphyses; (**4a**,**4b**) apical and lower cells; (**4c**) showing dichotomous branching. All photos from the type (S-F50933) (**2e**,**3a**–**3c**,**3f**,**4a**–**4c**) in Congo Red pre-treated with KOH or (**3d**,**3e**) in MLZ pre-treated with KOH.

**Figure 3:**
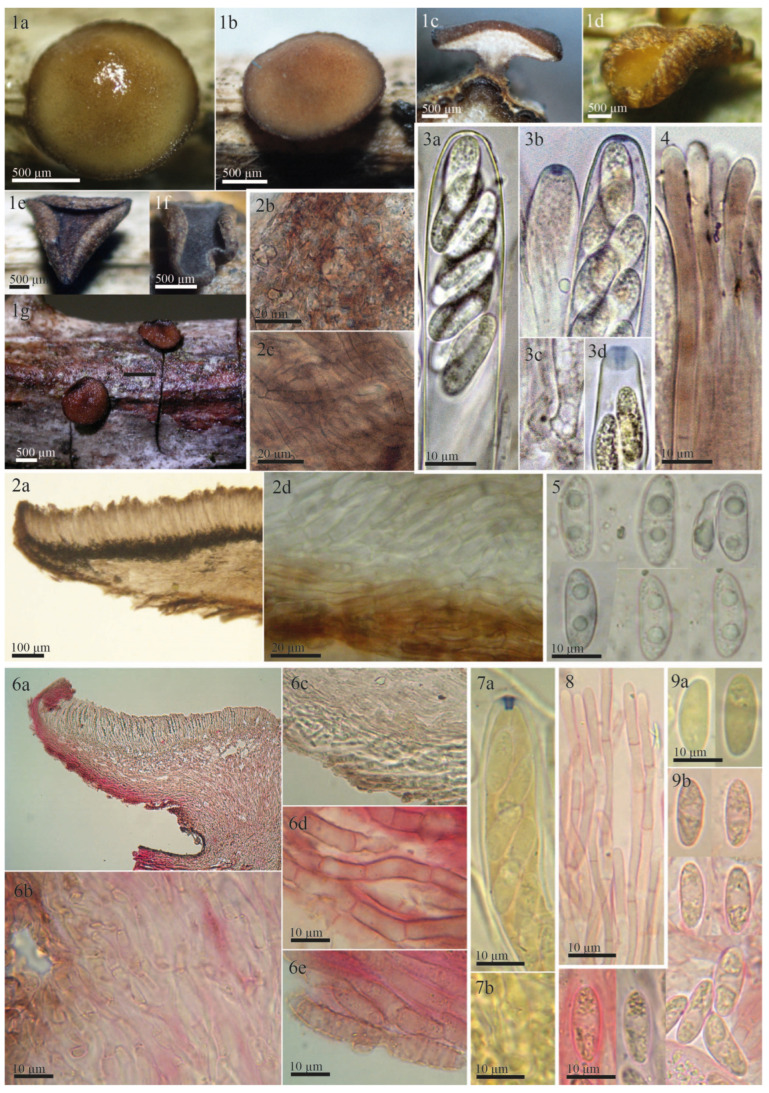
Morphological details of other collections of *Rutstroemia vagabunda* examined by the authors or B. Perić: (**1**–**5**) from fresh (living) collections (**1a**–**1g**,**2b**,**2c**,**3a**–**3d**,**4**: from B.P. C7D-10-12-17, published in [23] as *R. fruticeti*; **2a**,**2d**,**5**: from H.B. 9502); (**6**–**9**) microscopic details from the type specimen of *R. fruticeti* (NY-01167778); (**1a**–**1d**) fresh apothecia (**1c** in transversal section); (**1e**,**1f**) dry apothecia; (**1g**) rehydrated apothecia; (**2a**–**2d**,**6a**–**6e**) transversal section showing details of the excipulum; (**2b**) octahedral crystals on surface of excipulum; (**2c**,**6d**,**6e**) cortical cells with irregular patches of dark exudate that produce a banded aspect; (**2d**,**6b**,**6c**) ectal excipular cells embedded in refractive hyaline gel; (**3a**) living ascus with biseriate spore arrangement; (**3b**,**3d**,**7a**) asci with amyloid ring of *Sclerotinia*-type (3 in IKI, 7 in KOH+MLZ, living in **3b** right); (**3c**,**7b**) ascus base arising from croziers; (**4**,**8**) paraphyses (living in **4**, with brown vacuolar content); (**5**,**9a**,**9b**) ascospores (living in **5**), note different guttule pattern among (**3**,**5**,**9**).

**Figure 4:**
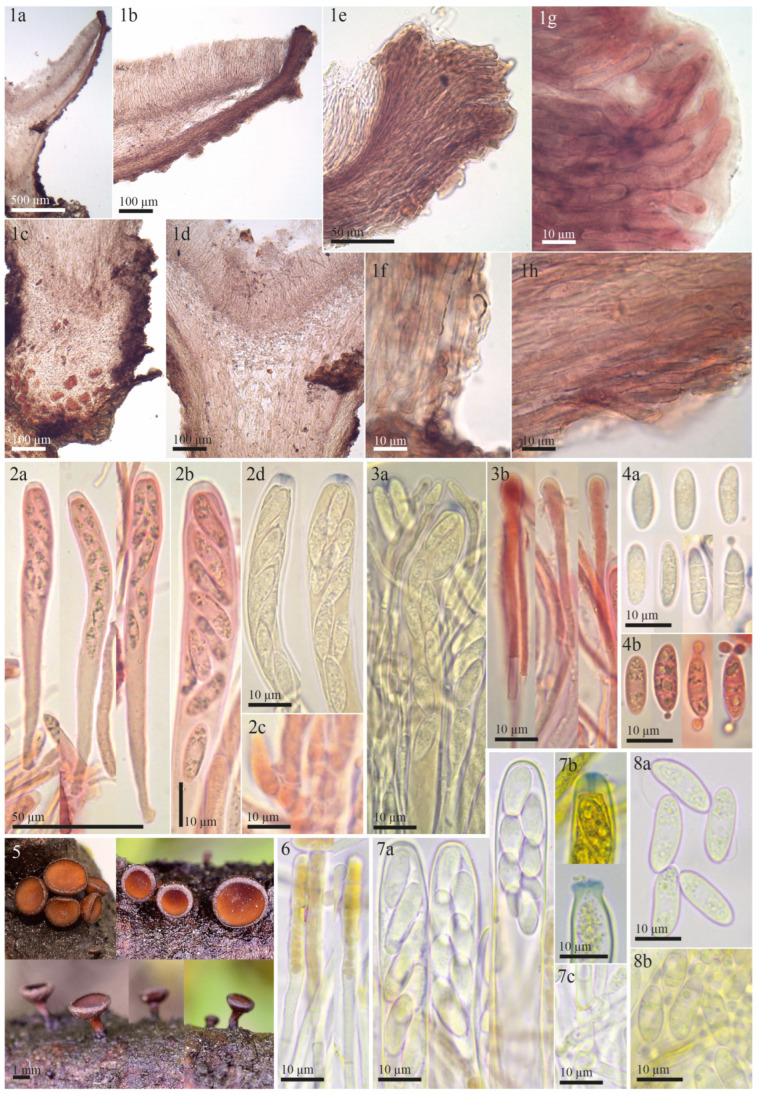
Morphological details for *Rutstroemia juniperi* examined by the authors or E. Stöckli: (**1**–**4**) microscopic details of the holotype collection from Sweden (NY-01167800); (**5**–**8**) fresh collections from Italy and Switzerland showing living elements (**5**,**7c**,**8b**: E.S. 2018.53, **6**,**7a**,**7b**,**8a**,**8b**: 26.06.2018); (**1a**–**1d**) transversal section showing details of the excipulum; (**1e**–**1h**) ectal excipulum with cortical cells with irregular patches of dark exudate that produces a banded aspect; (**1g**) ectal excipular cells at margin embedded in refractive hyaline gel; (**2a**,**2b**) dead asci; (**7a**) living asci; (**2c**,**7c**) asci arising from croziers; (**2d**,**7b**) asci with *Sclerotinia*-type of amyloid ring (**2d** KOH-pretreated, **7b** in IKI); (**3a**,**3b**) dead paraphyses; (**6**) living paraphyses with yellowish vacuolar content; (**4a**,**4b**) dead ascospores at different stages of maturity, overmature with septa and conidia; (**8a**) living mature ascospores (multiguttulate); (**8b**) living conidia formed on overmature ascospores.

**Figure 5:**
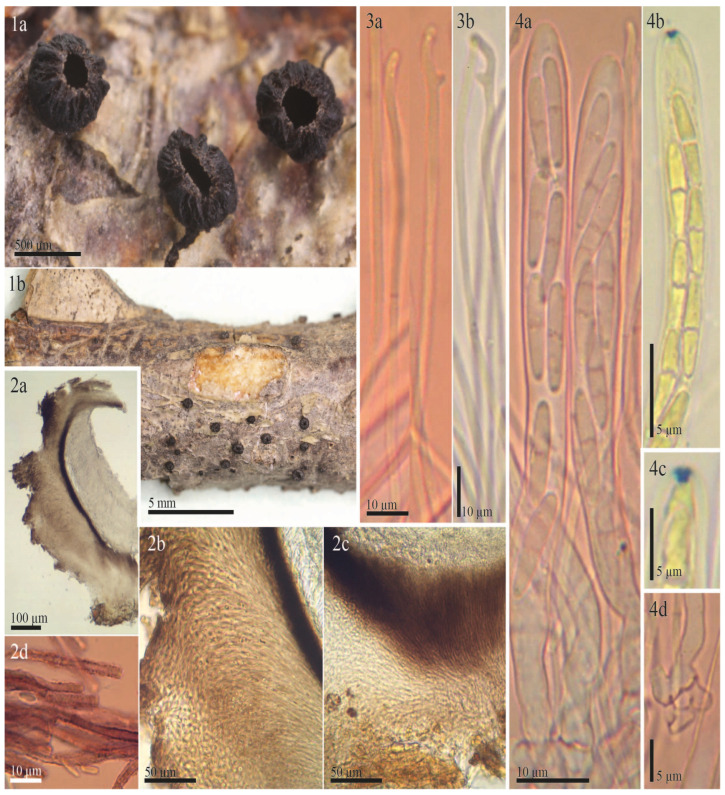
Morphological details for the syntype of *Rutstroemia longiasca* (on *Rosa spinosissima*): (**1a**,**1b**) rehydrated apothecia; (**2a**–**2d**) excipular tissues in transversal section; (**2b**) ectal excipulum at upper flank; (**2c**) excipulum near base; (**2d**) cortical cells embedded in refractive gel and with irregular patches of dark exudate on the walls; (**3a**,**3b**) paraphyses in Congo Red pretreated with KOH, note the partly bifurcate apices; (**4a**) asci in Congo Red pretreated with KOH; (**4b**,**4c**) asci in MLZ pretreated with KOH, with details of amyloid apical ring; (**4d**) ascus arising from croziers. All photos from Cav., F. Longob. Exs. 223 (FH).

**Figure 6:**
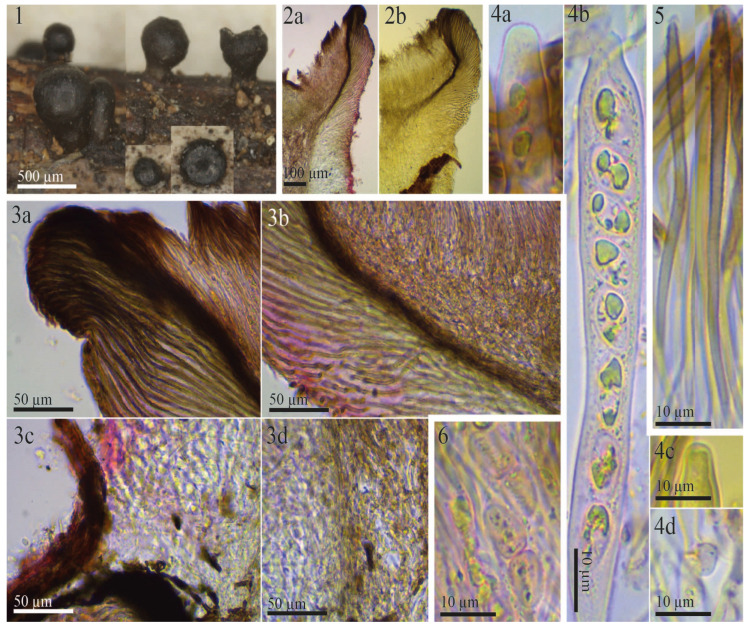
Morphological details for the syntype of *Rutstroemia urceolus* (from Switzerland): (**1**) rehydrated apothecia; (**2a**,**2b**) excipular tissues in transversal section; (**3a**,**3b**) ectal and medullary excipulum at margin and upper flank; (**3c**,**3d**) excipulum near base; (**4a**,**4b**) submature asci in Congo Red pre-treated with KOH, note immature ascospores with their lipid content; (**4c**) apex of immature ascus in Melzer’s reagent (pretreated with KOH); (**4d**) ascus base arising from crozier; (**5**) paraphyses in Congo Red (pretreated with KOH), note the thick dark wall in the apical cell; (**6**) overmature ascospores with septum and strong constriction (in KOH+CR). All photos from Fungi Rhenani Exsiccati 2474 (FH).

The examined type specimen of *Tympanis vagabunda* from S was a single, partly corticated twig with only three mature apothecia. *T. vagabunda* was collected in Italy (Europe), Sicilia, Manostalla, on an unidentified perennial herbaceous plant, IX.1878, leg. V. Beltrani (S-F50933) (Figure 1). The substrate is given in the protologue as “on dry, fallen twigs of *Pistacia terebinthus*, *Rosa* and *Rubus*”, which suggests that duplicates in other herbaria may exist in which the host may differ. The observed characteristics of the excipulum, asci, ascospores, and paraphyses revealed that *T. vagabunda* is indeed a *Rutstroemia* species (Figure 2). After reviewing the morphology, ecology, and distribution of all published species of *Rutstroemia*, we found only four species similar to *T. vagabunda*, namely: *R. longiasca*, *R. fruticeti*, *R. juniperi*, and *R. urceolus* (Figure 3, Figure 4, Figure 5 and Figure 6). For comparison among these species, see Table 2. Velenovský [22] described two more species on *Rosa* and *Rubus*: *Rutstroemia rosarum* Velen. and *R. rubi* Velen. These types were not studied; for details, see the discussion. In the following, we describe our study of the type of *Tympanis vagabunda*, which resulted in a new combination in the genus *Rutstroemia* (Figure 1, Figure 2 and Figure 3). We also provide illustrations of the type specimens of *R. longiasca*, *R. fruticeti*, *R. juniperi*, and *R. urceolus*, together with some collections studied in the living state (Figure 4, Figure 5 and Figure 6). 

***Rutstroemia vagabunda*** (Pass. and Beltrani) Quijada and Baral, comb. nov. (Figure 1, Figure 2 and Figure 3)

Mycobank number: MB 846748

Basionym: *Tympanis vagabunda* Pass. and Beltrani, Atti R. Acad. Lincei, Trans., sér. 3 7: 37 (1882)

≡ *Cenangium vagabundum* (Pass. and Beltrani) Sacc., Syll. fung. (Abellini) 8: 557 (1889)

= *Rutstroemia fruticeti* Rehm, Rabenh. Krypt.-Fl., Edn 2 (Leipzig) 1.3(lief. 39): 766 (1893) [1896]

≡ *Ciboria fruticeti* (Rehm) Sacc., Syll. fung. (Abellini) 11: 402 (1895)

Etymology: Passerini and Beltrani [6] did not explain why they chose “vagabunda” as the specific epithet (which means “wandering”). Rehm used “fruticeti”, named after the host, Rubus fruticosus.

Redescription of syntype of *Tympanis vagabunda*. **Apothecia** erumpent from greyish black stromatic lines on the host; rehydrated 0.8–1.4 mm in diam., up to 0.6 mm high, short stipitate, scattered to gregarious in groups of two or three, not or slightly gelatinous, shiny; closed when young, opening by a small pore, cupulate when mature; hymenium rehydrated deep red-brown (16.d.Red) to vivid deep red-brown (17.v.d.Red); margin convolute, entire and regularly lacerated by forming short, broad teeth, protruding up to 100 µm beyond disc; receptacle concolorous, surface slightly rough (Figure 2(1a–1c)). **Asci** cylindric-clavate, †(115)120–123(128) × (9)10–11.5(12) µm; 8-spored, spores 1–2-seriate, apical ring amyloid (blue, Sclerotinia-type) in MLZ or LUG with or without KOH pretreatment; arising from croziers (Figure 2(3a–3f)). **Ascospores** ellipsoid to cylindrical, †(7)10.7–11.5(15.1) × (3)3.9–4.2(4.5) µm, inequilateral to slightly curved (allantoid), non-septate, hyaline, thin-walled, with numerous 0.5–2 µm large LBs, multiguttulate (Figure 2(3b,3c)), no overmature spores or conidial formation observed (exceptionally 1-septate, Figure 2(3c)). **Paraphyses** cylindrical, uninflated above, hyaline, embedded in a hyaline gelatinous matrix, 3–4-septate; terminal cell †(21)26.5–37(49.5) × 2–3 µm, cell below †(15.5)17.5–24.5(30) × 1.5–2.5 µm; simple or branched, thin-walled, a few tiny drops present in each cell (Figure 2(4a–4c)). **Medullary excipulum** well developed, †70–120 µm thick, made up of non-gelatinized textura intricata, grayish pink (8.gy.Pink) to grayish red (19.gy.Red) in KOH, cells †16–30.5 × 3–4 µm, cell wall up to 0.5 µm thick. **Ectal excipulum** †80–145 µm thick at base and lower flank, †70–100 µm at upper flank and margin, composed of textura porrecta and differentiated into three layers (Figure 2(2c)). Outermost and innermost (Figure 2(2a,2b)) layer composed of sparse rows of loosely woven, horizontally oriented, greyish red (19.gy.Red) to medium reddish brown (43.m.rBr) cells of †(8.5)10.5–14.5(21) × (3.5)4–4.5(5) µm, embedded in refractive hyaline gel, gel of †0.5–2 µm thickness, cell wall up to 0.7 µm thick, incrusted with irregular patches of dark exudate that produce a banded aspect; intermediate layer similar but thicker, obliquely oriented, lighter pink-gray (10.pkGray) to grayish red (19.gy.Red), cells embedded in abundant refractive, hyaline gel, wavy and more frequently branched, †(13.5)14.5–18.5(22.5) × (3)4–5(6.5) µm, gel between cells †1–4 µm thick, cell wall smooth; outermost layer at upper flank and margin of nongelatinized wavy hyphae protruding to form short hair-like elements (Figure 2(2a,2b)). **Octahedral crystals** present in ectal excipulum, primarily in outermost layer at lower flank and base, 2.5–6.5 × 2.5–6 µm (Figure 2(2d)). **KOH-reaction** absent, no color changes and pigment released.

Material examined: ***Rutstroemia vagabunda***: EUROPE, ITALY, SICILIA: WSW of Palermo, NNE of Alcamo, Manostalla [as Monostalla, in sched. as Monastalla], ~60 m, on dry, fallen twigs of *Pistacia terebinthus*, *Rosa* and *Rubus* (only one branch in the package), September 1878, V. Beltrani (S-F50933, Syntype); GERMANY: close to Königstein, on dead stems of *Rubus fruticosus*, May 1877, W. Krieger (NY-01167778, Syntype); GERMANY: NORDRHEIN-WESTFALEN, N of Coesfeld, SE of Holtwick, NE of Höven, 103 m, on dead stems of *R. fruticosus*, 16 July 1991, K. Siepe (K.S. 91/24, H.B. 4488); BADEN-WÜRTTEMBERG, NW of Stuttgart, Feuerbach, Tannhäuserstr., 320 m, on dead stems of *R. fruticosus*, 29 September 1976, H.O. Baral (H.B. 854); BAYERN, OBERFRANKEN, Bayreuth, close to Stadtförsterei, 380 m, on dead stems of *R. fruticosus*, 16 June 1991, W. Beyer (H.B. 4489); FRANCE: PAYS-DE-LA-LOIRE, VENDÉE, WNW of La Tranche-sur-Mer, Plage de la Terrière, 10 m, on dry stems of *R. ¸fruticosus*, 3 June 2003, E. Weber (H.B. 7381); POITOU-CHARENTES, DEUX-SÈVRES, NW of Chizé, SSE of Villiers-en-Bois, Forêt de Chizé, 72 m, on dry stems of *R. fruticosus*, 27 April 2011, V. Baral (H.B. 9502); MONTENEGRO: Podgorica, NW of Gornji Martinići, ENE of Glizica, 220 m, on dry stems of *R. fruticosus*, 10 December 2017, D. Raspopovic, vid. B. Perić (B.P. Dgf/C7D-10-12-17). ***Rutstroemia juniperi***: EUROPE, SWEDEN: UPPSALA LÄN, Älvkarleö, NNE of Älvkarleby, peninsula Billudden, 1 m, on needles and twigs of *Juniperus communis*, 14 September 1969, K. and L. Holm (n. 17a-69, ex UPS, NY-01167800, ex Holotype); GERMANY: BADEN-WÜRTTEMBERG, WSW of Münsingen, SW of Gomadingen, Sternberg, 780 m, dry corticated twigs and needles of *J. communis*, 7 October 1989, H.O. Baral and O. Baral (H.B. 3871); SWITZERLAND, GRAUBÜNDEN, ESE of Davos, Flüelapass, Säss, 1964 m, on dry corticated twigs of *J. communis* ssp. *alpina*, 26 June 2018, J. Gilgen and E. Stöckli (unpreserved, voucher specimen from same place: 29.VI.2015, E.S. 2015.29); ITALY, TRENTINO-ALTO ADIGE, SW of Stelvio, Franzenshöhe, 2215 m, on dry corticated twigs of *J. communis* ssp. *alpina*, 16 August 2018, E. Stöckli (E.S. 2018.53). ***Rutstroemia longiasca***: ITALY: LOMBARDIA or REGGIO EMILIA, Apennine Mountains, Mt. Lesima, 800–1500 m, on dead branches of *Rosa spinosissima*, undated (autumn), R. Farneti (Cav. Fungi Longob. Exs. 223 in FH, Syntype). ***Rutstroemia urceolus***: SWITZERLAND: NEUCHÂTEL, around Neuchâtel, ~500 m, on rotten branches of *Rubus fruticosus*, undated, P. Morthier (Fuckel Fungi Rhen. 2474 in FH, Syntype). 

### 3.2. Phylogenetic Results

**Figure 7:**
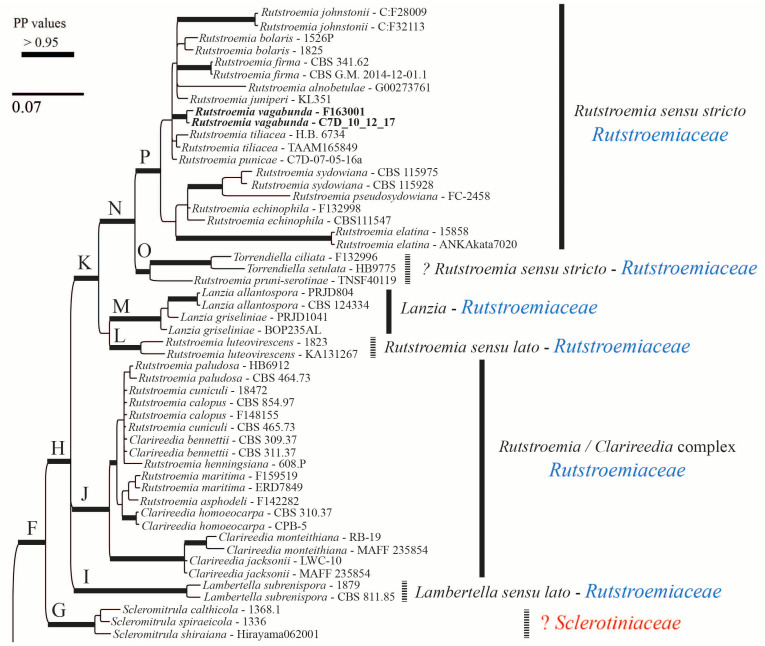
Bayesian majority-rule consensus tree based on concatenated ITS and LSU sequences. Bold branches are those which were well supported. Clades at generic or higher rank are numbered with letters on the left and their corresponding names of genera and families shown on the right. The two species treated in this article *(Rutstroemia vagabunda*, previously identified as *R. fruticeti*, and *R. juniperi*) are clustered in the core clade P of *Rutstroemia*. The new combination proposed (*R. vagabunda*) is in bold.

**Figure 8:**
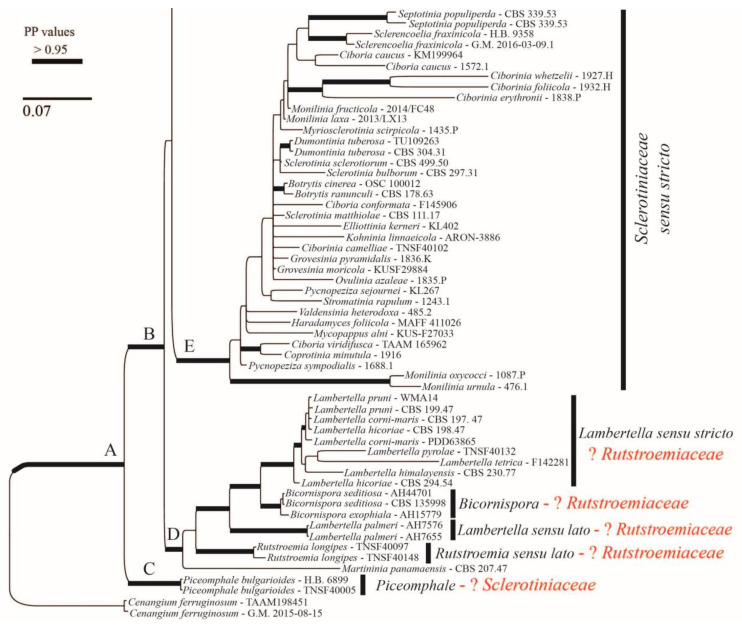
Continuation of Bayesian majority-rule consensus tree based on concatenated ITS and LSU sequences.

The alignment consisted of 1263 base pairs (83% of the first alignment length), of which 342 were parsimony-informative, 412 were variable, and 851 were constant. Here, we are using *s.s.* (*sensu stricto*) and *s.l.* (*sensu lato*) to discuss the clades that include the type species of the genus (*s.s.*) vs. those that do not include it (*s.l.*). The Bayesian tree is shown in Figure 7 and Figure 8. The family Rutstroemiaceae is resolved as polyphyletic with two main clades (Figure 7 and Figure 8: clades H and D) and Sclerotiniaceae *s.s.* nested between them. The genus *Rutstroemia* resolved as polyphyletic. Several supported clades can be differentiated (Figure 7 and Figure 8): clade P = *Rutstroemia s.s.*, which includes the type species *R. firma* (Pers.) P. Karst.; clade O = *R. pruni-serotinae* Whetzel and W.L. White, which is related to *Torrendiella* Boud. and Torrend; clade L = *R. luteovirescens* (Roberge ex Desm.) W.L. White; clade J = several species of *Clarireedia* L.A. Beirn, B.B. Clarke, C. Salgado and J.A. Crouch (with some species still under *Rutstroemia*); and clade D = *R. longipes* (Cooke and Peck) W.L. White, which is related to *Lambertella* Höhn. s.s. and *Bicornispora* Checa, Barrasa, M.N. Blanco and A.T. Martínez. The two sequences of *R. vagabunda* (in GenBank as *R. fruticeti*) are placed in clade P (Figure 7), but without a clear relationship to any other species. The *Rustroemia s.s* clade is the most diverse, with 12 species, followed by *Clarireedia* (Figure 7), with 11 different taxa that are partly conspecific; the necessary new combinations are proposed in another article in this journal (Baral et al. [29]).

## 4. Discussion

In the protologue of *Tympanis vagabunda*, Passerini and Beltrani [6] did not give morphological or biometric information about the excipulum and its cells. Their macroscopic description fits quite well with our observations obtained from the type specimen, as well as the morphology and biometry of asci and ascospores (Table 2). Although the authors stated that the asci were inamyloid, we discovered that they were amyloid (Figure 2(3d,3e)) with a *Sclerotinia*-type amyloid ring, as shown in Johnston et al. [12]. This type is found in most members of the Rustroemiaceae and Sclerotiniaceae. Although Passerini and Beltrani described the species in the genus *Tympanis*, it is unclear which classification system they followed. Their publication consists only of a species list with descriptions and some information about locality and hosts.

When Tode [30] erected the genus *Tympanis*, only *Tympanis saligna* Tode was included. The genus was described with globose to cup-shaped apothecia, which were gregariously clustered, leathery, black, and erumpent. There are no details of microscopic features, and the drawings only show the apothecia [30] (table IV, figures 37a,d–i) and anamorph [30] (table IV, figures 37b,c). Before Passerini and Beltrani [6] published *T. vagabunda*, only Fries [31] and Schweinitz [32] added species to the genus. *Tympanis* was conceived as something between pyrenomycetes and discomycetes, differentiated merely by its macroscopic features [1]. Fries [31] wrote “sporidia forma and numero varia, secedentia” which we translate as “spore shape and number variable, disintegrating”. This would agree with a microscopic characteristic of *Tympanis* currently circumscribed, which involves the presence of primary spores (ascospores) and secondary spores (ascoconidia). Each of the eight or four ascospores produce a usually large number of ascoconidia packed within a membrane to form 4–8 roundish balls within living asci ([33] figure 10a). In addition, in dead asci, it is possible to observe the succession of asci with eight ascospores through intermediate stages until they are filled with innumerable conidia [9]. These characteristics do not agree with Passerini and Beltrani’s [6] description of *Tympanis vagabunda* or our own observations (Figure 2(3a–3e)). Furthermore, all species recognized today in *Tympanis* differ in the construction of their ectal excipulum (plectenchymatous, *textura intricata-angularis*), paraphyses (moniliform), and inamyloid ascus apex [9]. Furthermore, the ecology of the two genera differs: *Tympanis* is a plant parasite with host specificity [1], whereas *Rutstroemia* is a saprophyte with rather high substrate specificity as well [27]. 

Our redescription of the type specimen of *Tympanis vagabunda* has clarified that this species should be treated in the genus *Rutstroemia*. Perić and Baral [23] provided an overview of the history of *Rutstroemia* and circumscribed the genus. Species in the genus *Rustroemia* can be characterized by: apothecia reddish brown or sometimes greenish yellow or olivaceous, discoid, short- to long-stipitate, erumpent from the host issue, with an ectal excipulum of prismatic or rarely angular cells, often enclosing a layer of gelatinized, long-celled hyphae, cortical and medullary hyphae roughened by a brown exudate that forms a banded aspect, asci with apical ring reacting deep blue in iodine (*Sclerotinia*-type), ascospores ellipsoid-cylindrical, often ± allantoid, with high or sometimes low lipid content, 1–3 septate when overmature, budding to produce globose conidia [23]. All of these features agree with our redescription of *Tympanis vagabunda* as presented above and in Figure 1 and Figure 2. For this reason, we conclude that the species described by Passerini and Beltrani [6] is indeed a *Rutstroemia*. Eighty-eight species names have been published in *Rutstroemia* [8]. Among them, only a few species share a similar morphology, ecology, and distribution with *Tympanis vagabunda* (Table 2).

In the protologue of *T. vagabunda*, Passerini and Beltrani [6] gave the host as “*Rosa*, *Rubus*, and *Pistacia*”. We were only able to locate one collection of this species (Figure 1), although it seems probable that duplicates exist in other herbaria. The examined type in herbarium S only contained a single twig, although the description mentions three different hosts. Microanatomical sections of the wood of this twig were interpreted by the second author as excluding any of the three cited host genera, as well as other woody Rosaceae, based on pores in a distinct radial arrangement instead of a ring- or scattered-pored arrangement. Therefore, we refrain from designating a lectotype here. Our interpretation of *R. vagabunda* as conspecific with *R. fruticeti* is based on the morphological similarities of these fungi. Because *R. juniperi* has a very similar morphology but strongly differs from *R. fruticeti* in DNA sequences currently available for comparison, it cannot be excluded that different species of *Rutstroemia* exist on angiosperms other than *Rubus*. 

*Rutstroemia fruticeti* is currently considered to be restricted to *Rubus fruticosus* agg. [23,25,34]. Its apothecia can vary in color from light brown to reddish or almost black depending on the age and degree of hydration (Figure 3(1a–1g), for further details about its features in the living state, see [23]). In our revision of *Tympanis vagabunda*, measurements and morphology were found to be consistent with *R. fruticeti* (Figure 1, Figure 2 and Figure 3, Table 2). All morphological features indicate that the type specimen of *R. fruticeti* (Germany), as well as recent collections from Germany and Montenegro (Figure 3), are conspecific with the type specimen of *T. vagabunda* (Italy) (Figure 1 and Figure 2). The shared characteristics can be summarized as follows: (1) reddish apothecia; (2) ectal excipulum composed of *textura porrecta* oriented horizontally and obliquely and differentiated into three layers, with octahedral crystals and cortical cells with irregular patches of dark exudate that have a banded aspect; (3) eight-spored asci with amyloid apical rings (*Sclerotinia*-type), arising from croziers; (4) ellipsoid-cylindrical, guttulate ascospores; and (5) cylindrical, apically uninflated paraphyses. Our biometric study of *T. vagabunda* (Table 2) also agrees with the type and recent collections of Perić and Baral [23] shown in Figure 3. Therefore, we conclude that *T. vagabunda* is conspecific with *R. fruticeti*. 

*Rutstroemia juniperi* (Figure 4) is very similar to *Rutstroemia vagabunda* (= *R. fruticeti*), but it grows on a gymnosperm host, *Juniperus*. Despite the very similar macro- and micromorphology and overlapping measurements for asci and ascospores (Table 2), the phylogenetic analyses by Pärtel et al. [14] and ours show that *R. vagabunda* and *R. juniperi* are not closely related and can be recognized as two species (Figure 7 and Figure 8). At least in this case, the host appears to be fundamental to differentiating the species (angiosperm vs. gymnosperm). In contrast, there are species in the genus with a similar host spectrum and distribution as *R. fruticeti* but with distinct morphological differences, such as *R. longiasca* and *R. urceolus* (Figure 5 and Figure 6). All three have been reported from Europe (Italy, Germany, Montenegro, and Switzerland,) on either *Rosa* or *Rubus* [23,24,25,27,28]. 

In his monograph of *Rutstroemia*, White [27] combined *Pyrenopeziza longiasca* Cav. with *Rutstroemia* and provided an extensive description and illustration. All the characters described by White agreed with our study of the type specimen (Figure 5, Table 2). The apothecia of *R. longiasca* are black and shallow-cupulate (Figure 5(1a,1b)); in transverse sections, we could differentiate the complex structure with a thick ectal excipulum with two distinct layers (Figure 5(2a–2c)); ascus and ascospore measurements by White [27] agree with our measurements (Table 2), although we observed some narrower asci. White [27] did not describe the amyloid reaction of the asci or the croziers (Figure 5(4b–4d)). Like Cavara [35], White described the ascospores as 1-septate, whereas we found they can be up to 3-septate (Figure 5(4a)). *R. longiasca* is differentiated from *R. vagabunda* by its black apothecia, smaller asci, and narrower ascospores (Table 2). A further striking difference lies in the “lack of oil globules” in the ascospores [27], which is confirmed here (Figure 5(4a)). 

The type specimen of *Patellea urceolus* Sacc. Also grows on *Rubus fruticosus*, as evidenced in [7] (p. 784) and [27] (p. 194). White combined the species in *Rutstroemia* and provided a very short description of the type specimen in FH, which lacks various information, such as ascus iodine reaction and ascospore contents, and mainly repeats the data of the protologue. On p. 229, he briefly mentioned its similarities to the protologue of *R. fruticeti*. Macroscopically, *R. urceolus* differs through its black apothecia when rehydrated (Figure 6(1)). Microscopically, the ascospores cannot be separated, their biometry strongly overlaps, and their shape and content are very similar: ellipsoid to cylindrical, 0–1-septate, with a high lipid content (Table 2, Figure 2 and Figure 6). Although the biometry of asci overlaps as well, the ascus apex of *R. urceolus* is thin-walled and inamyloid (Figure 6(4a–4c)), whereas that of *R. vagabunda* has a pronounced apical thickening with an amyloid ring (Figure 3(3b,3d,7a)). 

Although *Rutstroemia rubi* Velen. and *R. rosarum* Velen. share the same hosts (*Rubus* and *Rosa*, respectively) with *R. vagabunda*, we did not review the types of these species but rather relied on the published information available. *R. rubi* was considered a possible synonym of *R. fruticeti* by White [27], solely based on the protologues of the two species, which he stated to be known only from the type collections and the identical host, *Rubus fruticosus* (in *R. rubi*, also *Prunus spinosa*). However, *R. rubi* possesses prominent—though not further described—hairs on the receptacle and stipe [22], which Graddon [25] illustrated in detail based on a British collection on *Rubus fruticosus* referred by him to *R. rubi*. Spooner [36] examined this collection and probably correctly placed *R. rubi*, with hesitation, in synonymy with *Torrendiella ciliata* Boud. In contrast, White [27] pointed out the similarities among *R. rosarum*, *R. fruticeti*, and *R. longiasca*. Our studies of the type indicate that only *R. longiasca* could be conspecific with *R. rosarum*. Both species have asci shorter than 105 µm and ascospores narrower than 3.5 µm (*R. rosarum*: asci 60–90 × 8–10 µm, ascospores 12–15 × 3 µm [22,27]) and both occur on *Rosa*, whereas *R. vagabunda* (= *R. fruticeti*) clearly differs in its longer asci and wider ascospores (Table 2).

## 5. Conclusions

The genus *Rutstroemia* is polyphyletic based on ITS and LSU phylogenetic analyses and comprises several clades. Since White’s [27] monographic revision, the genus has not been thoroughly investigated, and many of its species still lack molecular data. *Rutstroemia vagabunda* is a saprobe found on *Rubus* in central and Mediterranean Europe. Whether it can also grow on *Rosa*, *Pistacia*, or other hosts remains to be clarified. Fresh collections on these diverse hosts are needed to clarify if this species also occurs on angiosperms other than *Rubus*. According to our results, the very morphologically similar *R. juniperi* can be differentiated from *R. vagabunda* only by growing on a gymnosperm (*Juniperus*) and by its DNA. More studies are needed to better understand the ecology of the species in the genus *Rutstroemia*, in particular their host specificity, to consider dividing the genus, and to clarify whether the family Rutstroemiaceae needs to be redefined, as it is currently para- or polyphyletic.

## Figures and Tables

**Table 1:** Samples used in this molecular study and their GenBank accession numbers and voucher information. Two fresh collections of *Rutstroemia vagabunda* (= *Tympanis vagabunda*), which were identified as *R. fruticeti*.

Species	ITS	LSU	Isolate	Host Species	Locality
*Bicornispora exophiala*	KF499363	N/A	AH15779	*Cytisus oromediterraneus*	Spain
*Bicornispora seditiosa*	KF499361	KF499361	AH44701	*Pistacia terebinthus*	Spain
*Bicornispora seditiosa*	KF499360	KF499360	CBS135998	*Acer monspessulanum*	Spain
*Botrytis cinerea*	DQ491491	AY544651	OSC 100012	N/A	N/A
*Botrytis ranunculi*	NR_164278	N/A	CBS 178.63	*Ranunculus abortivus*	New York
*Ciboria caucus*	MZ159549	N/A	KM199964	*Alnus glutinosa*	United Kingdom
*Ciboria caucus*	Z73766	Z73740	1572.1	*Salix caprea*	Norway
*Ciboria conformata*	KJ941075	KJ941057	F145906	*Alnus glutinosa*	Spain
*Ciboria viridifusca*	LT158429	KX090812	TAAM 165962	*Alnus* sp.	Estonia
*Ciborinia camelliae*	AB926074	AB926159	TNSF40102	N/A	Japan
*Ciborinia erythronii*	Z73767	Z73741	1932.H	*Erythronium* sp.	Canada
*Ciborinia foliicola*	Z80892	Z81404	1932.H	*Salix* sp.	Canada
*Ciborinia whetzelii*	Z73768	Z73742	1927.H	*Populus tremuloides*	Canada
*Clarireedia bennettii*	MF964321	N/A	CBS 309.37	N/A	United Kingdom
*Clarireedia bennettii*	MF964323	N/A	CBS 311.37	N/A	United Kingdom
*Clarireedia homoeocarpa*	MF964322	MH867420	CBS 310.37	N/A	United Kingdom
*Clarireedia homoeocarpa*	KF545313	N/A	CPB-5	*Festuca rubra*	United Kingdom
*Clarireedia jacksonii*	MF964320	N/A	LWC-10	*Agrostis stolonifera*	North Carolina
*Clarireedia jacksonii*	KF545301	N/A	MAFF 235854	*Agrostis stolonifera*	Japan
*Clarireedia monteithiana*	KF545306	N/A	RB-19	*Cynodon dactylon x transvaalensis*	Massachusetts
*Clarireedia monteithiana*	KF545305	N/A	MAFF 236938	*Cynodon dactylon*	Japan
*Coprotinia minutula*	Z81428/Z81429	Z81405	1916	N/A	Canada
*Dumontinia tuberosa*	LT158412	KX090843	TU109263	*Anemone nemorosa*	Estonia
*Dumontinia tuberosa*	MH855223	MH866674	CBS 304.31	N/A	New York
*Elliottinia kerneri*	LT158475	N/A	KL402	*Abies alba*	Switzerland
*Grovesinia moricola*	MG564341	N/A	KUSF29884	*Parthenocissus tricuspidata*	South Korea
*Grovesinia pyramidalis*	Z81433	Z81409	1836.K	*Juglans nigra*	USA
*Haradamyces foliicola*	AB329720	N/A	MAFF 411026	*Cornus florida*	Japan
*Kohninia linnaeicola*	AY236423	N/A	ARON-3886	*Linnaea borealis*	Norway
*Lambertella corni-maris*	MH856215	N/A	CBS 197. 47	*Malus sylvestris*	Switzerland
*Lambertella corni-maris*	MH921865	N/A	PDD63865	*Cyttaria* sp.	New Zealand
*Lambertella hicoriae*	KF545337	MH868882	CBS 294.54	N/A	Wisconsin
*Lambertella hicoriae*	MH856216	MH867746	CBS 198.47	*Carya ovata*	New York
*Lambertella himalayensis*	MH861053	MH872822	CBS 230.77	*Cassia siamea*	Burma
*Lambertella palmeri*	KF499364	KF499364	AH7576	*Quercus ilex*	Spain
*Lambertella palmeri*	KF499365	KF499365	AH7655	*Quercus ilex*	Spain
*Lambertella pruni*	DQ335471	N/A	WMA14	*Prunus persica*	Oregon
*Lambertella pruni*	MH856217	MH867747	CBS 199.47	*Prunus avium*	USA
*Lambertella pyrolae*	AB926081	N/A	TNSF40132	N/A	Japan
*Lambertella subrenispora*	KC533549	N/A	1879	*Aster ageratoides*	Japan
*Lambertella subrenispora*	KF545329	MH873604	CBS 811.85	*Aster ageratoides*	Japan
*Lambertella tetrica*	KJ941068	N/A	F142281	*Hedera helix*	Spain
*Lanzia allantospora*	AY755334	N/A	PRJD804	*Agathis australis*	New Zealand
*Lanzia allantospora*	AB926099	N/A	CBS 124334	*Agathis australis*	New Zealand
*Lanzia griseliniae*	AY755333	N/A	PRJD1041	*Griselinia littoralis*	New Zealand
*Lanzia griseliniae*	MH003473	N/A	BOP235AL	*Polylepis incana*	Ecuador
*Martininia panamaensis*	MH856219	MH867749	CBS 207.47	N/A	Panama
*Monilinia fructicola*	LT615175	LT615175	2014/FC48	*Prunus persica*	Hungary
*Monilinia laxa*	LT615173	LT615173	2013/LX13	*Prunus triloba*	Hungary
*Monilinia oxycocci*	Z73789	Z73754	1087.P	*Oxycoccus quadripetalus*	Norway
*Monilinia urnula*	Z73794	Z73758	476.1	*Vaccinium vitis-idaea*	Norway
*Mycopappus alni*	KC753529	KY696722	KUS-F27033	*Salix koreensis*	Korea
*Myriosclerotinia scirpicola*	Z81440	N/A	1435.P	*Scirpus lacustris*	Norway
*Ovulinia azaleae*	Z73797	Z73760	1835.P	*Rhododendron* sp.	N/A
*Piceomphale bulgarioides*	KJ941086	KJ941062	H.B. 6899	*Picea abies*	Switzerland
*Piceomphale bulgarioides*	AB926053	AB926122	TNSF40005	*Abies* sp.	Japan
*Pycnopeziza sejournei*	LT158443	KX090827	KL267	*Hedera helix*	France
*Pycnopeziza sympodialis*	Z81445	Z81418	1688.1	*Betula pubescens*	Norway
*Rutstroemia alnobetulae*	MW677580	N/A	G00273761	*Alnus alnobetula*	Switzerland
*Rutstroemia asphodeli*	KJ941085	KJ941065	F142282	*Asphodelus*	Spain
*Rutstroemia bolaris*	Z80894	Z81419	1526P	*Betula pubescens*	Norway
*Rutstroemia bolaris*	KC533546	N/A	isolate-1825	N/A	Norway
*Rutstroemia calopus*	KF545314	N/A	CBS 854.97	dead grass	Netherlands
*Rutstroemia calopus*	KF588373	N/A	F148155	*Festuca indigesta*	Spain
*Rutstroemia cuniculi*	KC533548	N/A	18472	N/A	United Kingdom
*Rutstroemia cuniculi*	KF588375	N/A	CBS 465.73	N/A	United Kingdom
*Rutstroemia echinophila*	KF588371	KJ941053	F132998	*Quercus ilex*	Spain
*Rutstroemia echinophila*	KF545332	N/A	CBS 111547	*Quercus castaneifolia*	Netherlands
*Rutstroemia elatina*	JF908711	N/A	15858	N/A	Italy
*Rutstroemia elatina*	MN263048	N/A	ANKAkata7020	*Abies nordmanniana*	Turkey
*Rutstroemia firma*	KF588369	MH869768	CBS 341.62	N/A	France
*Rutstroemia firma*	KT876987	KT876987	G.M. 2014-12-01.1	*Quercus* sp.	Luxembourg
*Rutstroemia henningsiana*	Z81442	Z81416	608.P	*Carex rostrata*	Norway
*Rutstroemia johnstonii*	LT158454	N/A	C:F28009	*Xenotypa aterrima*	Denmark
*Rutstroemia johnstonii*	LT158456	N/A	C:F32113	*Xenotypa aterrima*	Denmark
*Rutstroemia juniperi*	LT158465	N/A	KL351	*Juniperus communis*	Norway
*Rutstroemia longipes*	AB926073	AB926142	TNSF40097	*Daphniphyllum macropodum*	Japan
*Rutstroemia longipes*	AB926105	N/A	TNSF40148	N/A	Japan
*Rutstroemia luteovirescens*	KC533545	Z81412	1823	*Acer platanoides*	Norway
*Rutstroemia luteovirescens*	KR673723	N/A	KA131267	N/A	South Korea
*Rutstroemia maritima*	KJ941084	KJ941064	F159519	Unidentified grasses	Spain
*Rutstroemia maritima*	MT370345	N/A	E.R.D. 7849	*Iris germanica*	Spain
*Rutstroemia paludosa*	KF588376	N/A	H.B. 6912	*Juncus effusus*	Luxembourg
*Rutstroemia paludosa*	KF545316	N/A	CBS 464.73	*Symplocarpus foetidus*	New York
*Rutstroemia pruni-serotinae*	AB926083	AB926173	TNSF40119	*Prunus grayana*	Japan
*Rutstroemia pseudosydowiana*	AB904500	N/A	FC-2458	*Quercus crispa*	Japan
*Rutstroemia punicae*	MK501758	MK501758	C7D-07-05-16a	*Punica granatum*	Montenegro
*Rutstroemia sydowiana*	AY853238	N/A	CBS115975	N/A	Netherlands
*Rutstroemia sydowiana*	KF545330	N/A	CBS115928	N/A	Netherlands
*Rutstroemia tiliacea*	LT158423	KX090808	HB6734	*Tilia* sp.	Germany
*Rutstroemia tiliacea*	LT158428	N/A	TAAM165849	*Tilia* sp.	Estonia
*Rutstroemia vagabunda*	KF588370	N/A	F163001	*Rubus* sp.	Spain
*Rutstroemia vagabunda*	MK501759	MK501759	C7D-10-12-17	*Rubus* sp.	Montenegro
*Sclerencoelia fraxinicola*	KT876983	KT876983	H.B. 9358	*Fraxinus excelsior*	Germany
*Sclerencoelia fraxinicola*	MH194576	MH194576	G.M. 2016-03-09.1	*Populus tremula*	Luxembourg
*Scleromitrula calthicola*	Z80887	Z81422	1368.1	*Iris pseudacorus*	Norway
*Scleromitrula shiraiana*	AY789408	AY789407	Hirayama062001	N/A	N/A
*Scleromitrula spiraeicola*	Z81448	Z81424	1336	*Filipendula ulmaria*	Norway
*Sclerotinia bulborum*	MH855218	MH866668	CBS 297.31	N/A	Indiana
*Sclerotinia matthiolae*	MF964314	N/A	CBS 111.17	*Matthiola vallesiaca*	Switzerland
*Sclerotinia sclerotiorum*	MH856725	MH868246	CBS 499.50	*Linum usitatissimum*	Netherlands
*Septotinia podophyllina*	MH101502	MH101505	CBS 318.37	*Podophyllum peltatum*	New York
*Septotinia populiperda*	MH101503	MH101506	CBS 339.53	*Populus* sp.	Germany
*Stromatinia rapulum*	Z73801	Z73763	1243.1	*Polygonatum multiflorum*	Norway
*Torrendiella ciliata*	KC412008	KJ627220	F132996	*Quercus ilex*	Spain
*Torrendiella setulata*	KF588367	KJ941052	H.B. 9775	*Acer spicatum*	Canada
*Valdensinia heterodoxa*	Z81447	Z81423	485.2	*Vaccinium myritillus*	Norway
*Cenangium ferruginosum*	LT158471	KX090840	TAAM198451	*Pinus nigra*	Montenegro
*Cenangium ferruginosum*	KY462796	KY462796	G.M. 2015-08-15.1	*Pinus sylvestris*	Luxembourg

**Table 2:** Comparison among protologue, our reexamination of types (in bold), and some other collections with available data. All ascus and ascospores measurements refer to dead cells (except for spores of Kummer [24] and perhaps Graddon [25]).

Species	Author/s	Country	Host	Apothecia Color	Asci		Ascospores	
					Length	Width	Length	Width
*R. fruticeti*	Perić and Baral (2017) [23]	Montenegro	*Rubus*	Yellowish cinnamon to dark-brown	100–120	9–12	13–16	4–5
	Kummer 2002 [24]	Germany		Ochre-brown	105–160	11–17	13.5–18	5–7
	Graddon 1979 [25]	United Kingdom		Chocolate-brown	115	11	15.5–17	5–6
	**Our revision**	Germany		**?**	**116–128**	**9–11.5**	**9.9–15**	**4.5–6.3**
*R. juniperi*	Holm and Holm (1977) [26]	Sweden	*Juniperus*	Reddish brown	110–130	9	12–18	5.5
	**Our revision**			**Reddish brown**	**106–138**	**9.5–14.5**	**11.4–15.9**	**3.8–5.5**
*R. longiasca*	White 1941 [27]	Italy	*Rosa*	Black-brown	80–100	7–8	12–14	3–3.5
	**Our revision**			**Black**	**77–104**	**5.4–7.2**	**9.9–16.3**	**2.1–3.4**
*R. urceolus*	White 1941 [27]	Switzerland	*Rubus*	Black	90–100	9–10	16	8
	Höhnel (1907) [28]			Black	130–140	9–10	12–16	8
	**Our revision**			**Black-brown**	**130–145**	**8.5–10**	**10.5–17**	**4.8–6.8**
*R. vagabunda* (*≡ Tympanis vagabunda*)	Passerini and Beltrani 1883 [6])	Italy	*Rosa*, *Rubus*, *Pistacia*	Red-brown	112–125	10–12	12	5
	**Our revision**			**Red-brown**	**115–128**	**9–12**	**7–15**	**3–5.5**

## Data Availability

All sequences used in this study are available in GenBank.
